# Laparoscopic and robotic urology surgery during global Pandemic COVID-19

**DOI:** 10.1590/S1677-5538.IBJU.2020.S113

**Published:** 2020-07-27

**Authors:** Hamilton C. Zampolli, Alejandro R. Rodriguez

**Affiliations:** 1 Instituto do Câncer Arnaldo Vieira de Carvalho São PauloSP Brasil Instituto do Câncer Arnaldo Vieira de Carvalho, São Paulo, SP, Brasil; 2 Rochester General Hospital Urology Associates of Rochester RochesterNew York US Urology Associates of Rochester, Rochester General Hospital, Rochester, New York, US

**Keywords:** Laparoscopy, COVID-19 [Supplementary Concept], Robotic Surgical Procedures

## Abstract

Known laparoscopic and robotic assisted approaches and techniques for the surgical management of urological malignant and benign diseases are commonly used around the World. During the global pandemic COVID-19, urology surgeons had to reorganize their daily surgical practice. A concern with the use of minimally invasive techniques arose due to a proposed risk of viral transmission of the coronavirus disease with the creation of pneumoperitoneum. Due to this, we reviewed the literature to evaluate the use of laparoscopy and robotics during the pandemic COVID-19. A literature review of viral transmission in surgery and of the available literature regarding the transmission of the COVID-19 virus was performed up to April 30, 2020. We additionally reviewed surgical society guidelines and recommendations regarding surgery during this pandemic. Few studies have been performed on viral transmission during surgery. No study has been made regarding this area during minimally invasive urology cases. To date there is no study that demonstrates or can suggest the ability for a virus to be transmitted during surgical treatment whether open, laparoscopic or robotic. There is no society consensus on restricting laparoscopic or robotic surgery. However, there is expert consensus on modification of standard practices to minimize any risk of transmission. During the pandemic COVID-19 we recommend the use of specific personal protective equipment for the surgeon, anesthesiologist and nursing staff in the operating room. Modifications of standard practices during minimally invasive surgery such as using lowest intra-abdominal pressures possible, controlled smoke evacuation systems, and minimizing energy device usage are recommended.

## INTRODUCTION

The COVID-19 pandemic has been impacting our planet in an unprecedented way. Health care systems in several countries have collapsed in the face of a highly transmissible virus, potentially lethal and still poorly understood in its pathophysiology. Since the first cases of the disease caused by SARS-CoV-2, in the beginning of December 2019, not only medical practice has changed, but also the bases of social interaction, professional activity and the global economy have been hit hard.

In Urology, as in other specialties, surgeries have been reduced basically to emergencies. Elective surgeries for benign pathologies have been summarily postponed and elective oncological surgeries have been recommended in selected cases of pathologies with greater aggressiveness such as radical cystectomy for muscle-invasive or very high-risk non muscle-invasive bladder cancer; retroperitoneal lymph node dissection for testicular cancer; radical nephrectomy for cT3 tumors; nephroureterectomy for upper tract urothelial cancers; radical orchiectomy for testicular cancer and adrenalectomy for specific aggressive adrenal cancer pathology. Radical prostatectomy for high-risk prostate cancer and partial nephrectomy for ≥ cT1b renal tumors should be performed in centers located in areas not severely hit by the pandemic where the resources available are sufficient (1, 2).

COVID-19 pandemic has therefore, affected and will continue to influence how surgeons will approach the patient care peri-operatively. A risk-benefit assessment of each patient undergoing surgery should be performed based on the urgency of the surgery and the risk of viral illness and transmission. Among surgeons worldwide, a concern with the use of minimally invasive techniques (laparoscopic and robotic) has been raised due to a proposed risk of viral transmission of the COVID-19 with the creation of pneumoperitoneum.

Our understanding of the process of viral transmission in surgery is limited. The virus responsible for COVID-19 (SARSCoV-2) belongs to the subgroup of coronaviruses that include the severe acute respiratory syndrome coronavirus (SARSCoV) and the Middle East respiratory syndrome coronavirus (MERS-CoV). Although very similar to these viruses, COVID-19 appears to be highly contagious due to its longer latency period. The only current known modality of transmission of the COVID-19 virus is through respiratory droplet transmission (3–5).

The mechanism for successful transmission is thought to be two-fold: human to human when the infected person coughs or exhales droplets that reach the other persons nose, mouth, or eyes to enter their respiratory tract; or contaminated surfaces when larger droplets produced from the infected person are spread onto surrounding surfaces and another person touches these contaminated surfaces and then touches their eyes, nose, or mouth.

Another proposed mechanism has been suggested, although sufficient evidence is lacking, that an aerosolizing procedure on an infected person creates smaller droplets from the respiratory tract that are thought to be able to reach up to 1-m distance reaching another person's nose, mouth, or eyes.

However, since the only proven mode of transmission of COVID-19 is through respiratory droplets, the risk of transmission from the abdomen is unclear (6).

Considering the hypothesis of a potential risk of exposure of the surgical staff to particles that could transmit COVID-19, during laparoscopic and robotic surgery, we reviewed the literature to evaluate the safety of these minimally invasive techniques during the global pandemic COVID-19.

### Evidence

In pure laparoscopic or robotic assisted surgery, part of the technique is the establishment and maintenance of an artificial pneumoperitoneum; with this comes the risk of aerosol exposure for the operating room team. Electrosurgical devices, including electrocautery and vessel-sealing tools, are now widely used intraoperatively for hemostasis. These devices enable surgeons to perform minimally invasive surgery, however, the surgical smoke that arises from electrosurgical devices may expose the surgical team to potentially harmful chemicals, viruses and viable cells (7–11). Therefore, acquiring an infectious disease from surgical smoke represents a potential health hazard.

Ultrasonic scalpels or electrical equipment commonly used in laparoscopic surgery can produce large amounts of surgical smoke, and in particular, the low-temperature aerosol from ultrasonic scalpels cannot effectively deactivate the cellular components of virus in patients. Li et al., found that after using electrical or ultrasonic equipment for 10 minutes, the particle concentration of the smoke in laparoscopic surgery was significantly higher than that in traditional open surgery (12). The reason may be that due to the low gas mobility in the pneumoperitoneum, the aerosol formed during the operation tends to concentrate in the abdominal cavity. Sudden release of trocar valves, non-air-tight exchange of instruments, or even small abdominal extraction incisions can potentially expose the health care team to the pneumoperitoneum aerosol.

Zhang et al. demonstrated the high prevalence of SARS-CoV-2 in stools (2, 13), but also the suggestion that the virus can be found in the gastrointestinal mucosa. Thus, despite the lack of evidence to demonstrate or refute the viral transmissibility from the gastrointestinal tract, a threat that the virus can be transmitted from the abdomen exists. And some have theorized that the environment created by pneumoperitoneum for laparoscopy creates a relatively stagnant heated volume of gas in the abdominal cavity which may subsequently allow for a concentrated aerosolization of the virus. Thus, it is hypothesized that sudden bursts of this pneumoperitoneum from trocar valves during exchange of instruments or during the venting of trocars can allow for transmission of the virus (6).

Many studies have reported the presence of other viruses in surgical smoke. Kwak et al., presented the first report of hepatitis B virus isolated from laparoscopic surgical smoke, successfully detected using a high efficiency collector and nested PCR, and higher concentration of surgical smoke particles in laparoscopic compared to open surgery (1, 13–15). Zheng et al. postulated a potential risk of SARS-CoV-2 diffusion during all minimally invasive procedures with possible subsequent infection of medical personnel working in operating rooms (15).

Although it is feasible for aerosols and microparticles to be released into the operating room during minimally invasive surgery, there is no scientific evidence so far, that particularly in the case of COVID-19, could demonstrate a greater risk of contamination of the surgical team by this route, and, to date, there are no reports of contamination of the surgical team by the coronavirus during minimally invasive surgery.

In fact, pure laparoscopic surgery, or robot assisted, seems to be safer, favoring both patients and the professional team that assists them. Although the risk of exposure to aerosols appears to be higher in minimally invasive surgery than in open surgery, the latter has an extremely higher risk of spreading micro and macroparticles, blood and tissues to the surgical team.

Actually, the use of laparoscopy during this pandemic can contribute to decreased length of stay as compared with open surgery as well as minimizing the need for medical treatments, and in turn increasing availability of beds, a limited resource. Laparoscopy is less traumatic compared with a laparotomy, and in the case of a patient infected with COVID-19, a minimally invasive operation as compared with an open procedure might result in improved survival and faster recovery. Laparoscopy allows for a self-contained operative field with less and possibly no spillage of fluids and tissues, thus decreasing any risk to the operative staff. For this reason, in the 1990s during the acquired immunodeficiency syndrome (AIDS) epidemic, laparoscopic surgery was strongly encouraged over open surgery in patients infected with the human immunodeficiency virus (HIV) (16, 17).

Finally, laparoscopic surgery, and in particular robotic surgery, allow for the staff and surgeon to be remote from the patient and from each other minimizing the risk of transmission of virus not only from the patient to the staff but also from operative staff infecting each other, as operative staff are in much closer proximity to each other and to the patient during open operations. Thus, as reviewed here, the benefits of laparoscopy that we have promoted and valued for many years can still provide a benefit even during the current pandemic and may even offer other benefits to this specific situation we may not have otherwise appreciated (6).

Few studies have been performed on viral transmission during surgery, but to date there is no study that demonstrates or can suggest the ability for a virus to be transmitted during surgical treatment whether open or laparoscopic. There is no consensus, among societies, on limiting or restricting laparoscopic or robotic surgery; however, there is expert consensus on the modification of standard practices to minimize any risk of transmission (6).

## CONCLUSIONS

Considering the data available so far, laparoscopic or robotic surgery can be considered safe procedures and should be performed, observing some modifications in order to reduce any possible risk to the surgical team. Despite very little evidence to support viral transmission through minimally invasive surgery, it is common sense to adopt measures that minimize any risk making modifications to surgical practice such as the use of smoke evacuation, lowering the pneumoperitoneum as low as possible and minimizing energy device usage among other measures to minimize operative staff exposure to aerosolized particles (Table 1 and 2) (11). Avoid intraoperative smoke formation by lowering electro cautery power settings, using bipolar electro cautery, using electro cautery or ultrasonic scalpels parsimoniously. More extensive use of sutures and clips in the operating room is recommended. Special attention must be paid when removing trocars at the end of a procedure, using suction to remove smoke and aerosol. Limit the smoke dispersal or spillage from trocars by lowering the pneumoperitoneum pressure. Usage of pressure-barrier insufflator systems that maintain a forced-gas pressure barrier at the proximal end of the trocar might be of benefit (2).

**Table 1:** Expected debris from the various categories of energy devices used in the abdomen.

Surgical Device	Plume
Ultrasonic Scalpel	0.35 - 6.5 microns
Laser ablation	0.3 microns
Electro cautery	< 0.1 microns

**Table 2:** Filtration devices for laparoscopy and robotics.

Device	Filter (microns)	Efficiency (%)
N95	0.3	95
HEPA	0.3	99.7
ULPA	0.05	99.9
ConMed PlumePort ActiV	0.1	99.9
Stryker PureView Active Plume	0.1	99.9
Stryker Pneumoclear Insufflator	0.051	99.9
ConMed AirSeal System	0.01	99.9

Finally, the need of appropriate personal protective equipment (PPE) should be reinforced. Nasopharyngeal samples should be obtained and tested (PCR) for all patients undergoing surgical procedures. Only negative COVID patients should undergo surgery. Positive COVID patients should be deferred until the patient has recovered from the disease and has tested negative. Positive COVID patients, under emergency scenarios, should be treated as much as possible in a conservative approach and only taken to surgery if the case is life threatening, since the mortality rate in these cases is as high as 20% (18).

## RECOMMENDATIONS

**Prevention and management of aerosol dispersal:**

- During operations, instruments should be kept clean of blood and other body fluids. Special attention should be paid to the establishment of pneumoperitoneum, hemostasis, and cleaning at trocar sites or incisions to prevent any gush of body fluid caused by air leakage or uncontained laparotomy incisions.
- Once ports are placed, they should not be vented if possible.
- The insufflator should be “on” before the new port valve is opened to prevent gas from back flowing into the insufflator.
- Liberal use of suction devices to remove smoke and aerosol during operations, and especially, before converting from laparoscopy to open surgery or any extra-peritoneal maneuver.
- Avoid using 2-way pneumoperitoneum insufflators to prevent pathogens colonization of circulating aerosol in pneumoperitoneum circuit or the insufflator. It's recommended using a closed circuit with smoke evacuation device with high-efficiency particle air (HEPA) or ultra-low particulate air (ULPA) filters or best available equivalent substitute (6, 15). (Table-2 and Figures 1A–C)
- All pneumoperitoneum should be safely evacuated from the port attached to the filtration device before closure or trocar removal, specimen extraction, or conversion to open.
- Suture closure devices that allow for leakage of insufflation should be avoided. The fascia should be closed after desufflation.

**Figures - 1:**
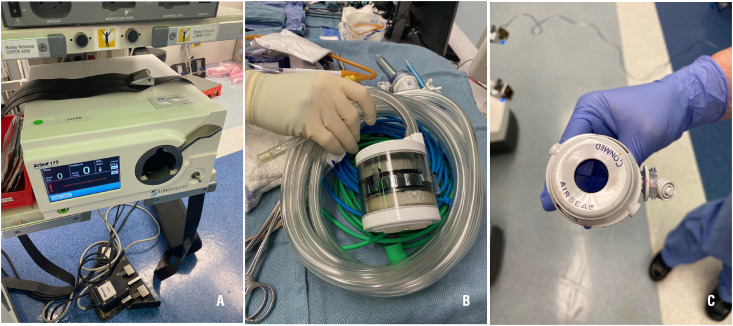
A, B, and C: Conmed® Airseal System.

(1A: Insufflator; 1B Filter and tubing; 1C Special Conmed® Trocar)

**Management of artificial pneumoperitoneum:**

- Keep intraoperative pneumoperitoneum pressure and CO2 ventilation at the lowest possible levels, since many emergency and non-emergency cases can be performed with an insufflation pressure of 12 mmHg or lower.
- Reduce the Trendelenburg position time as much as possible (2, 6, 15).
- At the conclusion of the operation to desufflate the abdomen use a smoke evacuation device or suction substitute (6).

**Operation techniques:**

- Minimize the use of energy devices during procedures when possible. When energy is needed, avoid the ultrasonic scalpel and lower energy settings to minimize surgical smoke (6, 15).

Surgery should be performed by an experienced laparoscopic or robotic surgeon to minimize length of surgical time as much as possible.

**Modifications for Robotic Surgery:**

- Use the same insufflators and smoke evacuation systems. Additional precautions to take with robotic surgery to avoid leakage from trocars include:
- Always using the trocar reducers in 12-mm trocars when inserting 8-mm or 5-mm instruments through the 12-mm trocars. Since the robotic ports and reducers are 8 mm, there is still potential leakage of pneumoperitoneum with 5-mm instruments. Thus, the use of laparoscopic 5-mm instruments through even the 8-mm trocars should perhaps be minimized if possible (6).
- Clean the console and the eyepiece, before and after using the system.

**Operating staff protection:**

- Best efforts must be made to raise awareness of the occupation protection on operating staffs, including surgeons, anesthetists, nurses and all possible transiting persons in the OR.
- Correct 2-way protective apparel (goggles, visor, mask, and body protective garb) should be routine.
- When engaging a suspected or diagnosed patient, tertiary dress code should be applied according to the protocols which also include strengthening OR ventilation and installing air purification equipment (2, 6, 15).
